# *Orangutan*: An R Package for Analyzing and Visualizing Phenotypic Data in the Context of Species Descriptions and Population Comparisons

**DOI:** 10.1002/ece3.73111

**Published:** 2026-02-20

**Authors:** Javier Torres

**Affiliations:** ^1^ School of Biological Sciences University of Nebraska‐Lincoln Lincoln Nebraska USA

**Keywords:** mensural variables, meristic variables, populations comparisons, species diagnosis, systematics, taxonomy

## Abstract

Phenotypic characters have long been central to species diagnosis and remain indispensable even in the age of genomics. However, phenotypic datasets are often complex—spanning dozens of traits of varying types and units, with correlated variables and unbalanced sampling—posing challenges for robust, reproducible analysis. Existing software solutions are fragmented, usually requiring labor‐intensive workflows across multiple tools and manual steps, which undermines reproducibility and hinders comparisons across studies. To address these methodological and practical challenges, I introduce *Orangutan*, an R package designed to provide a reproducible, easy‐to‐implement framework for comparing groups using mensural and meristic data. *Orangutan* integrates statistical analysis and visualization for species diagnosis and population comparisons within a single workflow. The package streamlines the identification of diagnostic, nonoverlapping traits between species, while enabling rigorous assessment of both individual and multivariate trait differences in overlapping traits. Core features include optional allometric correction to remove size effects, optional outlier removal, automated selection of appropriate univariate tests with post hoc comparisons, and integrated multivariate analyses. All outputs, including tables and publication‐ready figures, are generated with minimal coding, ensuring accessibility and standardization. Empirical validation with real‐world datasets—including animal and plant species—demonstrates that *Orangutan* robustly identifies diagnostic traits, reveals both subtle and clear group differences, and achieves high classification accuracy with phenotypic data alone. By automating and unifying key analytical steps, *Orangutan* promotes reproducibility, transparency, and efficiency in phenotypic research. This package could empower researchers in taxonomy, ecology, and evolutionary biology to adopt quantitative good practices for species diagnoses, facilitating comparative studies and advancing methodological standards in morphological data analysis. *Orangutan* is freely available as open‐source software with comprehensive documentation to facilitate broad adoption.

## Graphical abstract

*Orangutan* is an R package that streamlines the analysis of phenotypic data for species diagnosis and group comparisons using mensural and meristic traits. It automates key analytical steps—including size correction, statistical testing, and multivariate analysis—while producing standardized outputs and publication‐ready visualizations. This tool enhances reproducibility and accessibility in morphological research across taxonomy, ecology, and evolutionary biology.

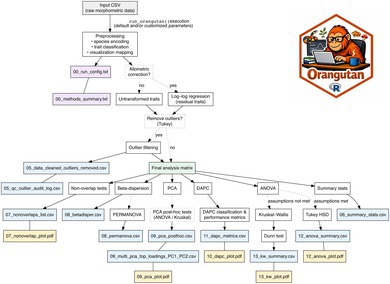

## 1 Introduction

Phenotypic characters have long been the cornerstone of taxonomic practice, serving as the basis for species diagnoses and revealing observable boundaries between species (Balakrishnan 2005). In fact, for practical and historical reasons most species have been described primarily on morphological grounds, even though genetic data are nowadays almost mandatory to delimit species. Phenotypic data remain indispensable for taxonomy, complementing molecular evidence and often revealing diagnostic differences that genetic data alone cannot capture (Cadena and Zapata 2021).

Phenotypic datasets for ecology and systematics are inherently complex, often including dozens of measurements per specimen and spanning diverse units (lengths, angles, indices, counts). In many cases trait values covary (e.g., allometric size effects), so one must normalize or apply allometric corrections to compare shape independently of body size. Data are often uneven—for example, few specimens per species but many characters or unbalanced group sizes. Small sample sizes combined with large numbers of variables invoke the “curse of dimensionality,” weakening statistical power and requiring feature selection or dimension‐reduction (e.g., Principal Components Analysis—PCA). In practice, rigorous phenotypic analysis typically demands preprocessing (e.g., standardizing variables, applying size corrections), diagnostic trait discovery, and multivariate statistics to summarize variation.

Unfortunately, the software tools for phenotypic analyses are fragmented. Researchers often cobble together custom workflows using general statistics packages or multiple specialized tools. The result is laborious preprocessing (e.g., spreadsheet edits, separate script for each analysis), ad hoc statistical tests (Analysis of Variance, PCA, etc.), and piecemeal visualization—a process that weakens reproducibility and makes it difficult to compare results across studies. At present, GroupStruct2 (Chan and Grismer 2025) is the only dedicated tool developed specifically to facilitate phenotypic data analysis for species diagnosis, providing a user‐friendly graphical interface that integrates statistical analyses and visualization. Here I introduce *Orangutan*, an R package with a similar overarching goal but designed for cases that require programmatic control. *Orangutan* enables standardized, end‐to‐end analytical workflows to be executed automatically across multiple group contrasts within a dataset, ensuring invariant preprocessing, statistical inference, and visualization, exact reproducibility through version‐controlled code, and seamless integration into larger analytical pipelines, while retaining simple and accessible execution for end users.

**FIGURE 1:**
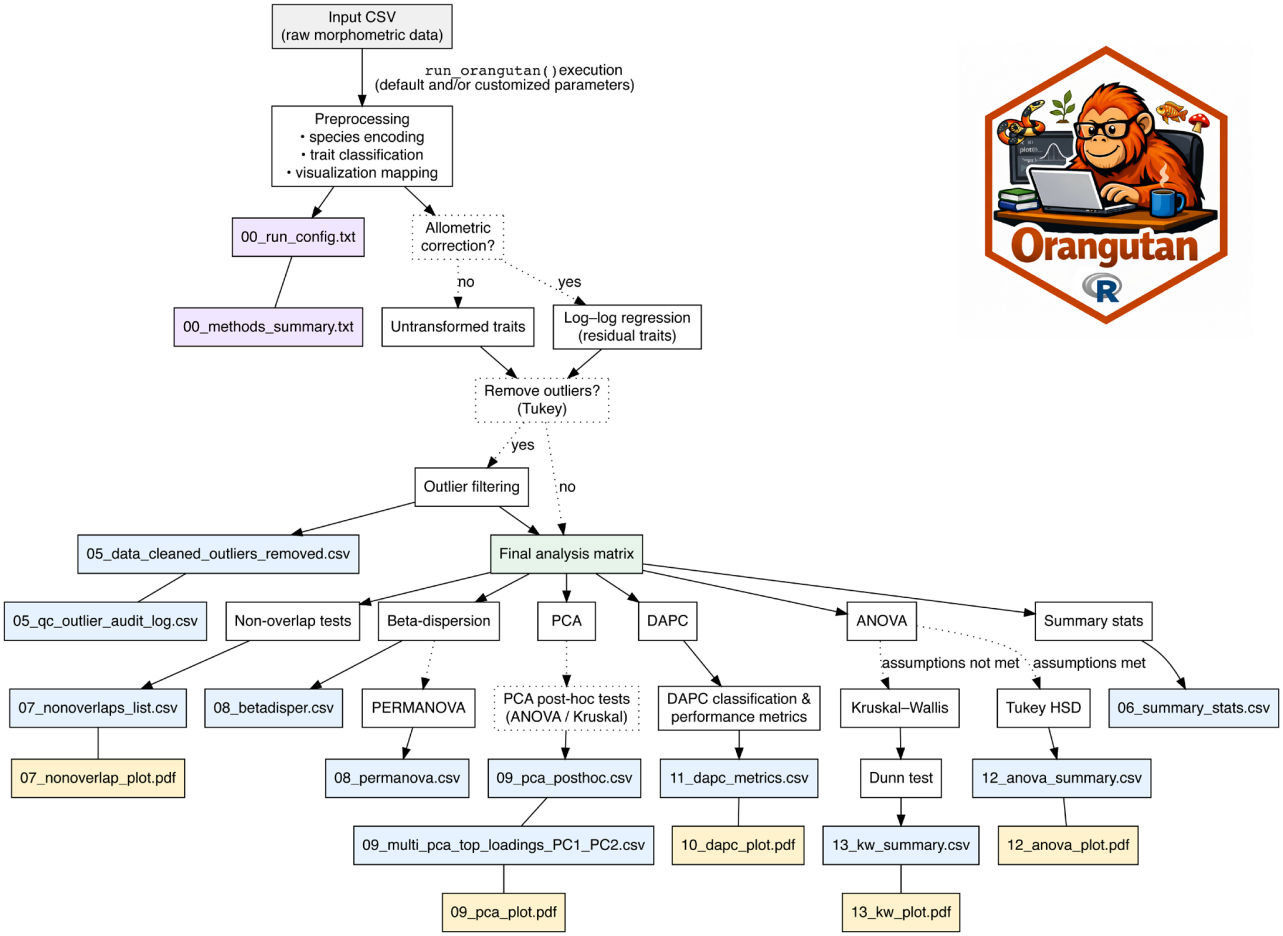
Diagram of the *Orangutan* phenotypic analysis workflow for species diagnosis. White boxes represent analytical steps, proceeding from a raw morphometric input matrix through preprocessing to univariate and multivariate analyses. Dotted boxes and arrows indicate optional or conditional steps that are executed only when relevant criteria are met. The green box denotes the final analysis matrix used for all downstream analyses. Purple boxes represent provenance and configuration outputs generated at run initialization, documenting analysis parameters and methodological settings. Blue boxes represent exported tabular outputs (CSV), whereas pastel boxes represent exported graphical outputs (PDF).

*Orangutan* provides an end‐to‐end toolkit for phenotype‐based species analysis: it uses a spreadsheet of mensural and meristic variables and identifies nonoverlapping traits—an important criterion for diagnosing species in the field. It also includes multivariate methods, such as PCA and Discriminant Analysis of Principal Components (DAPC), to explore the overall structure of phenotypic variation. For individual traits, *Orangutan* implements standard univariate statistical tests such as ANOVA or Kruskal–Wallis. All analyses are integrated with publication‐ready visualizations, enabling users to efficiently generate informative and standard figures for their papers. By combining these steps into a reproducible workflow (Figure 1), *Orangutan* streamlines the process of comparing populations using phenotypic data, including diagnosing and describing species.

In this manuscript I present *Orangutan*'s statistical and implementation framework in detail and demonstrate its utility with empirical case studies. I first overview the methods and choices behind *Orangutan*'s analyses, then illustrate how the package can be applied to real taxonomic datasets. My goal is to empower researchers with a flexible, reproducible toolkit for phenotype‐based species diagnosis and to promote and democratize good practices in morphological data analysis. By good practices, I refer to two complementary components of phenotypic analyses. The first concerns the quantitative treatment of phenotypic variation and species boundaries, including (i) explicit statistical comparison of within‐ and between‐group variation rather than reliance on qualitative diagnoses alone (Chan and Grismer 2021; Valcárcel and Vargas 2010), (ii) the use of multivariate statistical frameworks that account for covariance among traits and maximize discriminatory power among groups (Jombart et al. 2010; Lever et al. 2017; Valcárcel and Vargas 2010), and (iii) quantitative assessment of support for species boundaries, including trait differentiation, overlap, and classification performance. The second concerns transparent and reproducible analytical practice, emphasizing clearly documented assumptions, parameter choices, and fully reproducible workflows (Wilkinson et al. 2025). *Orangutan* operationalizes these principles in a unified, accessible framework, lowering the barrier for taxonomists and systematists to apply these practices routinely in their own research.

## 2 Methods

*Orangutan* is a package written in R (R Core Team 2024), designed to facilitate the statistical analysis and visualization of phenotypic data in the context of species descriptions or, in general, population comparisons. The package streamlines phenotypic data handling, allowing users to load structured datasets, conduct statistical comparisons, and generate informative visualizations with minimal coding effort (e.g., full execution in a single function—*run_orangutan*). All visualizations are saved with publication quality, for example, vector‐based PDF format with 7.5 in. width and 6 in. height.

*Orangutan* supports a multi‐faceted approach to assessing phenotypic distinctiveness between groups. First, it enables the identification of nonoverlapping traits—traits whose distributions do not overlap between groups—offering a clear and often desirable form of phenotypic diagnosability when describing species (Braby et al. 2024). Second, the package allows users to explore differentiation based on entire suites of traits by conducting multivariate analyses, including clustering and classification. These analyses assess whether groups form distinct clusters in multivariate space and whether individuals can be accurately assigned to their respective groups based on phenotypic characters alone. Third, *Orangutan* facilitates the detection of statistically significant differences in individual trait values, even when their distributions overlap. Such statistically significant differences provide evidence of average‐level divergence that may indicate underlying genetic, ecological, or evolutionary separation.

Key functionalities include identifying and removing outliers (optionally), detecting nonoverlapping traits, applying allometric corrections (optionally), and automatically selecting and conducting appropriate parametric or nonparametric tests for univariate analyses. By integrating these components, *Orangutan* enables researchers to robustly and reproducibly analyze phenotypic variation, assess different dimensions of phenotypic distinctness, and identify traits consistent with species differentiation with ease. The package is freely available at GitHub and CRAN with detailed instructions for installation and execution.

### 2.1 Dependencies and Input Data

The *Orangutan* package depends on a suite of R libraries for statistical analysis, data manipulation, and visualization. These include *adegenet* (Jombart 2008), used for multivariate analysis, specifically implementing DAPC. *vegan* (Oksanen 2025) implements multivariate distance‐based analyses, including PERMANOVA and assessment of multivariate dispersion (PERMDISP). *dunn.test* (Dinno 2024) performs post hoc pairwise comparisons following Kruskal–Wallis tests to identify significant differences among taxa. *dplyr* (Wickham et al. 2023) facilitates data manipulation tasks such as filtering, selecting, sorting, and summarizing datasets, supporting efficient data cleaning and transformation. *ggplot2* and *ggpubr* (Kassambara 2023; Wickham 2016) create layered visualizations including PCA plots, DAPC plots, and violin and boxplots. *multcompView* (Graves et al. 2024) converts post hoc test results into letter‐based groupings that are overlaid on plots to highlight significant differences. *tidyr* (Wickham and Girlich 2022) reshapes data for analysis and plotting, ensuring datasets are in the correct format. RColorBrewer (Neuwirth 2002) supplies distinct color palettes, improving the interpretability of species‐specific plots.

As data input, *Orangutan* expects phenotypic data in a structured format as a comma‐separated values (CSV) file where the first column—species—contains taxon or population designations, and the remaining columns contain meristic and mensural variables. Upon loading, the data undergoes removal of unwanted columns, such as generic index columns, and sorting alphabetically by species names to ensure consistency across downstream analyses. To further enhance the user experience, *Orangutan* assigns colors to taxa dynamically using the RColorBrewer palettes, with the number of colors tailored to the number of taxa in the dataset. If the number of taxa exceeds the palette's default limit (e.g., 12 colors for Paired), the color scheme is extended by cycling through available colors.

I used three empirical datasets to validate *Orangutan* by running them to test the different elements of the package. One dataset comprises four species of anole lizards and one putative hybrid category (Torres 2024). Another dataset comprises nine species of anole lizards (Torres et al. 2025). A third dataset is the “Iris” dataset included in R. It comprises three plant species and the main difference from the anole datasets is that the “Iris” dataset does not require allometric correction. The anole datasets can be downloaded from https://github.com/metalofis/Orangutan‐R/tree/main/example_datasets.

### 2.2 Optional Transformations and Filtering

#### 2.2.1 Allometric Transformation of Mensural Traits

To ensure that trait comparisons are biologically meaningful, *Orangutan* includes an optional allometric correction step in which users specify a size‐related variable as a scaling proxy. This variable represents a standard body or structure size against which the other size variables are scaled. For animals, the scaling proxy might be snout–vent length in amphibians and reptiles, or total body length in fishes. For plants, it could be stem height or inflorescence axis length, depending on the structure being analyzed.

This step is essential for distinguishing shape‐based differences from size‐related effects, particularly when comparing mensural traits in taxonomic and comparative morphological analyses. The allometric adjustment process begins by identifying the column representing the scaling variable. Both the scaling variable and the rest of the mensural variables are then log‐transformed to linearize the allometric relationships, following standard approaches to size–shape separation in morphometric analyses (Mosimann 1970; Thorpe 1975). A linear regression is performed for each mensural trait against log‐transformed scaling variable, and the residuals from this regression represent size‐independent trait variation and are retained as allometry‐adjusted variables (Mosimann 1970; Nakagawa et al. 2017). The final dataset consists of these residuals, which are combined with species classification, while non‐mensural traits remain unchanged. This allometric transformation ensures that subsequent statistical analyses focus on shape differences rather than size variation, improving the reliability of taxa comparisons in systematic and comparative studies (Chan and Grismer 2021; Thorpe 1975).

#### 2.2.2 Outlier Removal

Phenotypic datasets used in species descriptions often contain extreme values that may arise from measurement error, specimen damage, transcription mistakes, or rare aberrant individuals. Such values can disproportionately influence summary statistics, univariate tests, and multivariate ordinations, particularly in datasets with small or unbalanced sample sizes. To address this issue while minimizing subjectivity, *Orangutan* implements an optional, rule‐based outlier removal procedure.

Outlier detection is performed within species (or population) rather than across the full dataset, ensuring that biologically meaningful interspecific differences are not mistakenly treated as aberrant values. For each numeric trait and each taxon independently, *Orangutan* applies a Tukey‐style criterion based on the interquartile range (IQR). In the classical Tukey method (Tukey 1977), Q1 and Q3 correspond to the first and third quartiles of the distribution; in *Orangutan*, users can instead define the effective threshold used to estimate this range. Values are flagged as outliers if they fall below Q1–1.5 × IQR or above Q3 + 1.5 × IQR, where Q1 and Q3 represent the lower and upper quantiles of the trait distribution within that taxon as determined by the user‐defined threshold. This criterion is widely used in exploratory data analysis and provides a conservative, distribution‐free approach that does not assume normality.

Outlier removal is applied sequentially by trait, meaning that for each variable, only the observations identified as outliers for that specific trait are removed, while the same individuals may be retained for other traits if they fall within acceptable ranges. This trait‐wise approach avoids wholesale exclusion of specimens based on a single extreme value and preserves as much biologically informative data as possible.

All removed observations are logged internally, including the taxon, variable name, original value, and outlier threshold, allowing users to fully audit the filtering process. If no outliers are detected for a given trait or dataset, the original data are retained unchanged. After outlier removal, species factor levels and associated color mappings are rebuilt to ensure consistency across downstream analyses and visualizations.

### 2.3 Descriptive Statistics

*Orangutan* computes summary statistics for each numeric trait by taxon, enabling reproducible and straightforward analysis. It automatically identifies numeric variables, adapting to different datasets without requiring user input. For each trait, it calculates the mean ± standard deviation, along with minimum and maximum values, summarizing results as “Mean ± SD (Min − Max)” (Table 1). Outputs are exported as a CSV file for easy reporting.

### 2.4 Identification of Nonoverlapping Variables

To evaluate species diagnosis, *Orangutan* identifies phenotypic traits with nonoverlapping value ranges between taxa pairs—useful for diagnosis. It checks whether a trait's range in one taxon does not intersect with that of another. When found, nonoverlapping traits are visualized with violin plots with embedded boxplots for mensural variables, whereas meristic variables are visualized using boxplots only. To report values, a table for the variable and taxa pairs that do not overlap is produced (Figure 1). If overlapping traits are not found, a message is provided. The overlapping traits are later tested in univariate analyses for statistical differences.

### 2.5 Multivariate Analyses

#### 2.5.1 PERMDISP and PERMANOVA

To formally test whether candidate taxa differ in multivariate trait space, *Orangutan* implements a permutational multivariate analysis of variance (PERMANOVA). This analysis evaluates whether the centroids of predefined taxa differ significantly in multivariate space based on a distance matrix derived from all numeric traits using Euclidean distances.

Prior to PERMANOVA, homogeneity of multivariate dispersions among taxa is assessed using a beta‐dispersion analysis (PERMDISP via *betadisper*) with permutation testing, which evaluates whether within‐group variances differ among groups. This step is essential because PERMANOVA assumes similar dispersion across groups; significant heterogeneity of dispersion can inflate Type I error rates and confound centroid‐based inference. Both overall and pairwise permutation tests of dispersion (999 permutations) are conducted.

Multivariate distances are calculated using Euclidean distance on the numeric trait matrix. A permutation test (999 permutations) is then applied to assess whether species identity explains a significant proportion of multivariate variation. PERMANOVA results are interpreted jointly with PERMDISP results: PERMANOVA outcomes are flagged as valid or potentially dispersion‐driven depending on whether beta‐dispersion is nonsignificant or significant, respectively, indicating whether detected multivariate differences reflect separation of group centroids rather than differences in within‐group dispersion, allowing explicit assessment of PERMANOVA validity based on dispersion structure.

Following formal hypothesis testing with PERMANOVA, principal component analysis (PCA) is used as an exploratory ordination method to visualize multivariate structure and summarize major axes of phenotypic variation.

#### 2.5.2 Principal Component Analysis (PCA)

To assess whether candidate species form distinct clusters in multivariate space, a PCA is performed using only the variables retained after filtering for non‐missing values and nonzero variance. PCA is used here as an exploratory ordination method to summarize overall patterns of phenotypic variation and to test the hypothesis that candidate taxa occupy distinct regions of multivariate trait space without imposing a priori group structure. By reducing dimensionality while retaining the major axes of variance, this analysis provides a quantitative framework for visualizing phenotypic similarity among individuals (Lever et al. 2017; Zhang and Castelló 2017). The PCA is conducted using the *prcomp* R function, with the data centered and scaled for standardizing (Bro and Smilde 2003). The first two principal components (PC1 and PC2) are extracted for further analysis. The contribution of each variable to PC1 and PC2 is assessed by calculating the absolute values of the loading scores, and the top ten variables contributing most to each principal component are identified. The explained variance for each principal component is computed by squaring the standard deviations of the principal components and dividing by the total variance, which is then expressed as a percentage on the plot. For visualization, convex hull polygons are created for any taxon declared by the user to delineate their phenotypic space.

To move beyond visualization and formally evaluate taxon‐level differences along PCA axes, post hoc statistical tests are applied to individual principal components. Principal components are retained for testing until cumulative explained variance reaches at least 90%. For each retained PC, normality (Shapiro–Wilk) and homoscedasticity (Bartlett's test) are assessed. PCs meeting parametric assumptions are analyzed using one‐way ANOVA followed by Tukey's HSD, whereas PCs violating assumptions are analyzed using Kruskal–Wallis tests followed by Dunn's post hoc comparisons with multiple‐test correction. Results from all PCA post hoc tests are summarized and exported as a table.

Because PCA is an unsupervised method that does not explicitly test group separability, *Orangutan* next applies a supervised discriminant framework (DAPC) to formally evaluate whether predefined candidate taxa can be statistically distinguished in multivariate trait space.

#### 2.5.3 Discriminant Analysis of Principal Components (DAPC)

Whereas PERMANOVA tests for overall multivariate differentiation among taxa, DAPC evaluates whether individuals can be reliably classified into predefined groups based on multivariate trait combinations. DAPC is a supervised multivariate method that combines PCA with Linear Discriminant Analysis by maximizing between‐group variation while minimizing within‐group variation, using predefined taxon assignments. This analysis tests the hypothesis that candidate species are phenotypically diagnosable and can be reliably discriminated based on multivariate trait combinations, beyond the exploratory patterns identified by PCA. By first reducing dimensionality with PCA and then applying linear discriminant analysis, DAPC avoids overfitting while providing an explicit framework for assessing group separation and classification accuracy (Jombart et al. 2010).

Using the *dapc* function from the *adegenet* package, dimensionality is first reduced using PCA, and the number of principal components retained is determined dynamically, retaining the minimum number of PCs required to explain at least 90% of the total variance. Linear discriminant analysis is then performed on the retained PC scores, with the number of discriminant axes (LDs) set to K − 1, where K is the number of predefined taxa. Classification robustness is assessed using a jackknife (leave‐one‐out) resampling procedure.

The coordinates of each individual along linear discriminant axes (LD1 and LD2 when applicable) are extracted and stored in a dataframe. The proportion of variance explained by LD1 and LD2 is calculated by dividing the corresponding eigenvalues by the sum of all eigenvalues. DAPC results are visualized using an ordination plot when two or more discriminant axes are available. In cases where only two taxa are present, DAPC yields a single discriminant axis (LD1), which is visualized using a one‐dimensional density plot rather than a two‐dimensional scatter plot. Like in the PCA plot, polygons can be overlaid on the DAPC scatter plot for any taxon to better visualize multivariate dispersion and group separation.

To assess classification performance, a confusion matrix is generated by comparing true taxon labels with predicted group assignments (Table 2). This matrix summarizes the results of jackknife‐resampled cross‐classification from the DAPC model. Rows show the a priori classifications, while columns show the model's predicted classifications. Diagonal elements indicate correctly classified individuals, and off‐diagonal elements indicate misclassifications. Elements above the diagonal represent false positives for the column class—cases where the model incorrectly assigned individuals from a given actual class to a different predicted class. Elements below the diagonal represent false negatives for the row class—cases where the model failed to assign individuals to their true class. In addition, individuals contributing to off‐diagonal elements of the confusion matrix are explicitly identified and summarized in a separate table. These misclassified specimens may reflect phenotypic overlap, conserved morphology, or potential taxonomic ambiguity, and thus provide biologically interpretable insight into the limits of multivariate discrimination among taxa.

To further quantify classification performance beyond raw counts, species‐wise diagnostic performance metrics are calculated from the confusion matrix, including sensitivity (true positive rate), specificity (true negative rate), and the True Skill Statistic (TSS). Sensitivity measures the proportion of individuals of a given taxon that are correctly classified, whereas specificity measures the proportion of individuals not belonging to that taxon that are correctly excluded. TSS combines both measures (sensitivity + specificity − 1) and provides a balanced assessment of classification performance that is robust to unequal group sizes and prevalence. TSS values range from −1 to +1, where values approaching +1 indicate strong discriminatory power, values near 0 indicate classification performance no better than random, and negative values indicate performance worse than random expectation (Allouche et al. 2006; Prescott et al. 2025).

### 2.6 Univariate Analyses

#### 2.6.1 ANOVA, Kruskal–Wallis and Post Hoc Tests

To test the hypothesis that individual phenotypic traits differ among candidate species or populations, one‐way Analysis of Variance (ANOVA) is performed for each variable. Prior to selecting the appropriate statistical test, assumption checks are conducted to evaluate whether parametric methods are justified. The assumptions of normality and homogeneity of variances are tested using the Shapiro–Wilk test and Bartlett's test, respectively. The Shapiro–Wilk test assesses whether trait values within groups are normally distributed, while Bartlett's test evaluates whether variances are equal across groups—both requirements for valid ANOVA inference. If both assumptions are met (*p*‐values > 0.05), ANOVA is conducted, and the results are interpreted based on the *F*‐statistic, degrees of freedom, and *p*‐value. For variables with significant ANOVA results (*p*‐value < 0.05), post hoc pairwise comparisons are performed using Tukey's Honestly Significant Difference (HSD) test, which controls the family‐wise error rate while identifying which specific group means differ. The results are summarized and visualized with violin plots and/or boxplots (for mensural and meristic variables, respectively), which are annotated with Tukey HSD labels to highlight significant differences. For variables failing ANOVA assumptions, the nonparametric Kruskal–Wallis test is used to compare traits between taxa, as it does not assume normality or homogeneity of variances and tests for differences in central tendency among groups. If the Kruskal–Wallis test is significant (*p*‐value < 0.05), pairwise post hoc comparisons are performed using Dunn's test with a Bonferroni correction, providing a conservative assessment of pairwise differences while controlling for multiple comparisons to prevent inflation of false positives (statistically significant differences that are detected even though no true biological difference exists). The results are summarized in a table and visualized with violin and/or boxplots annotated with Dunn's test labels.

#### 2.6.2 Summary Tables

Univariate analyses results are saved in summary tables. The ANOVA summary table reports, for each variable, the *F*‐statistic, *p*‐value, and whether the assumptions of normality and homogeneity of variances were met. The Kruskal–Wallis summary table reports, for each variable, the *p*‐value, Chi‐squared statistic, and any significant pairwise differences detected by Dunn's post hoc test. These tables enable a clear overview of which variables show significant differences among groups and by which statistical method the significance was determined.

## 3 Results

### 3.1 Descriptive Statistics

The function *compute_summary_stats* produces a CSV file with summary statistics for each species or population (Table 1), which is a common report in species descriptions to have a first, general idea of the variation among populations and whether there are differences among them. This function will dynamically identify numeric variables and run the summary statistics on only those when the dataset also contains categorical variables.

**TABLE 1:** Summary statistics (mean ± standard deviation and range) of a partial dataset from Torres et al. (2025). A: *Anolis*.

Species	Snout–vent length	Head length	Postmentals
*A. allisoni*	82.36 ± 7.1 (73–94.7)	26.48 ± 2.33 (24.7–31.8)	5.89 ± 0.78 (4–7)
*A. brunneus*	70.08 ± 1.42 (67–71.9)	21.22 ± 1.1 (19.7–23)	6.11 ± 0.33 (6–7)
*A. carolinensis*	59.3 ± 3.23 (55–63.3)	17.16 ± 1.31 (15.6–19.17)	6.12 ± 0.83 (5–8)
*A. fairchildi*	68.58 ± 1.85 (66–70.3)	20.86 ± 1.78 (18.9–22.4)	5.5 ± 1 (4–6)
*A. longiceps*	72.87 ± 4.42 (64.96–78.84)	22.96 ± 2.33 (20–27.21)	4.14 ± 0.38 (4–5)
*A. maynardii*	69.3 ± 0.71 (68.8–69.8)	21.5 ± 0.14 (21.4–21.6)	7 ± 1.41 (6–8)
*A. porcatus*	66.3 ± 8.03 (59.8–77.9)	21.45 ± 2.87 (18.79–25.4)	6 ± 0 (6–6)
*A. smaragdinus*	59.31 ± 3.66 (52.6–64.7)	16.19 ± 1.65 (13.3–18.9)	4.9 ± 1.2 (4–7)
*A. torresfundorai*	70.14 ± 5.19 (62.8–78)	22.42 ± 2.39 (18.7–26.08)	5.89 ± 1.05 (4–8)

### 3.2 Identification of Nonoverlapping Variables

**FIGURE 2:**
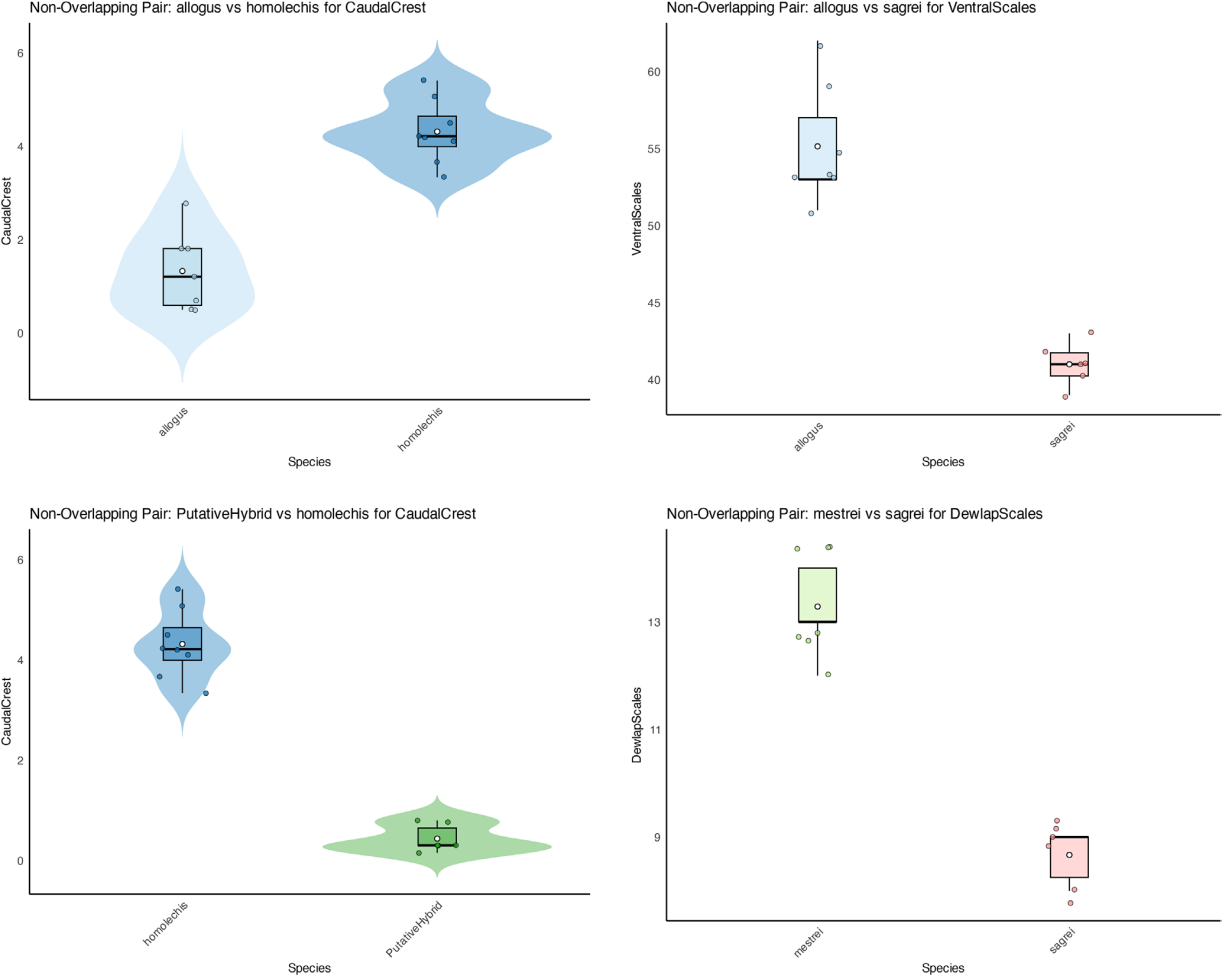
Sample of four nonoverlapping variables identified by *Orangutan* from the anole dataset of Torres (2024). Mensural variables are visualized using violin plots with embedded boxplots, whereas meristic variables are visualized using boxplots only, reflecting differences in data type and distribution. Boxplots show the interquartile range (25th–75th percentiles), white dot = mean, horizontal bar = median, and whiskers extending to 1.5× the interquartile range; points beyond this range represent outliers. Colors correspond to species or populations.

*Orangutan* identified variables that were diagnostic across species pairs and generated one violin and/or boxplot per variable per species pair (Figure 2). Colors were automatically assigned to each species or population. These plots summarize the distribution of each variable in cases in which species do not overlap in trait values. All non‐overlap cases are summarized in a CSV file.

### 3.3 Multivariate Analyses

Multivariate differentiation among species from the Torres (2024) dataset was significant (PERMANOVA: *F*_4,29_ = 12.62, *R* ^2^ = 0.64, *p* = 0.001). Tests of homogeneity of multivariate dispersions indicated no significant differences in within‐species variance (PERMDISP: *F*_4,29_ = 1.60, *p* = 0.185), indicating that PERMANOVA results reflect differences in group centroids rather than dispersion.

**FIGURE 3:**
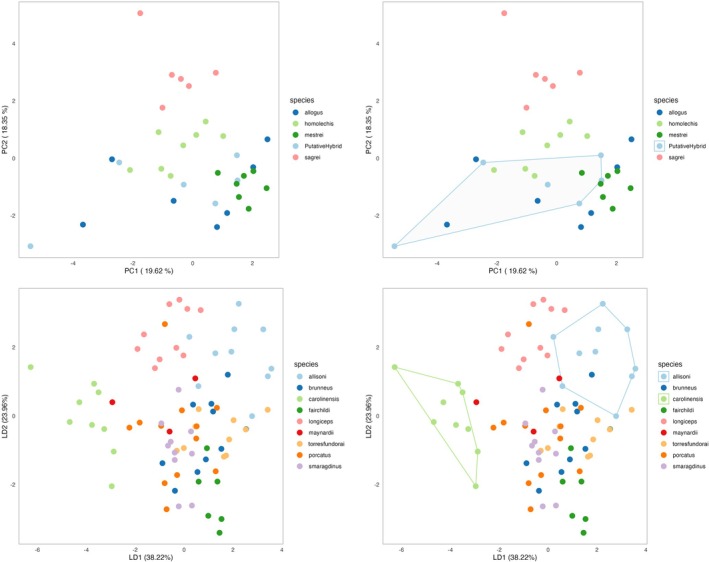
Multivariate analyses visualizations. PCA on the top using the Torres (2024) dataset. DAPC on the bottom using the Torres et al. (2025) dataset. The right panel depicts the option of declaring species to encircle using convex hulls, useful for emphasizing a given species/population.

The PCA and DAPC produced plots (Figure 3). A confusion matrix from the DAPC classification is also produced, showcasing the accuracy of taxon assignments (Table 2).

**TABLE 2:** Cross‐classification table resulting from the Torres et al. (2025) dataset: Jackknife‐resampled cross‐classification resulting from the linear discriminant analysis.

	alli	bru	car	fai	lon	may	por	sma	tor
alli	9	1	0	0	0	0	0	0	0
bru	0	8	0	1	0	0	1	0	0
car	0	0	9	0	0	0	0	0	0
fai	0	0	0	4	0	0	0	1	1
lon	0	0	0	0	10	0	0	0	0
may	0	0	1	0	0	2	0	0	0
por	0	0	0	0	0	2	6	2	2
sma	0	0	0	1	0	0	2	6	1
tor	0	1	0	0	0	0	3	0	6

*Note:* Rows represent a priori classification, while columns represent the model's classification. Values in the diagonal are correctly classified individuals, whereas values outside it are misclassifications, for an accuracy of 71.3% (57 of 80 individuals correctly classified). The elements above the diagonal are instances in which a sample from a row's actual class was incorrectly classified as a class to the right (a different predicted class). These are the false positives for the column class. The elements below the diagonal represent instances in which a sample from a column's predicted class was incorrectly classified as the class corresponding to the row. These are the false negatives for the row class. Species are *Anolis allisoni* (all), *A. brunneus* (bru), *A. carolinensis* (car), *A. fairchildi* (fai), *A. longiceps* (lon), *A. maynardii* (may), *A. porcatus* (por), *A. smaragdinus* (sma), and *A. torresfundorai* (tor).

### 3.4 Univariate Analyses

**FIGURE 4:**
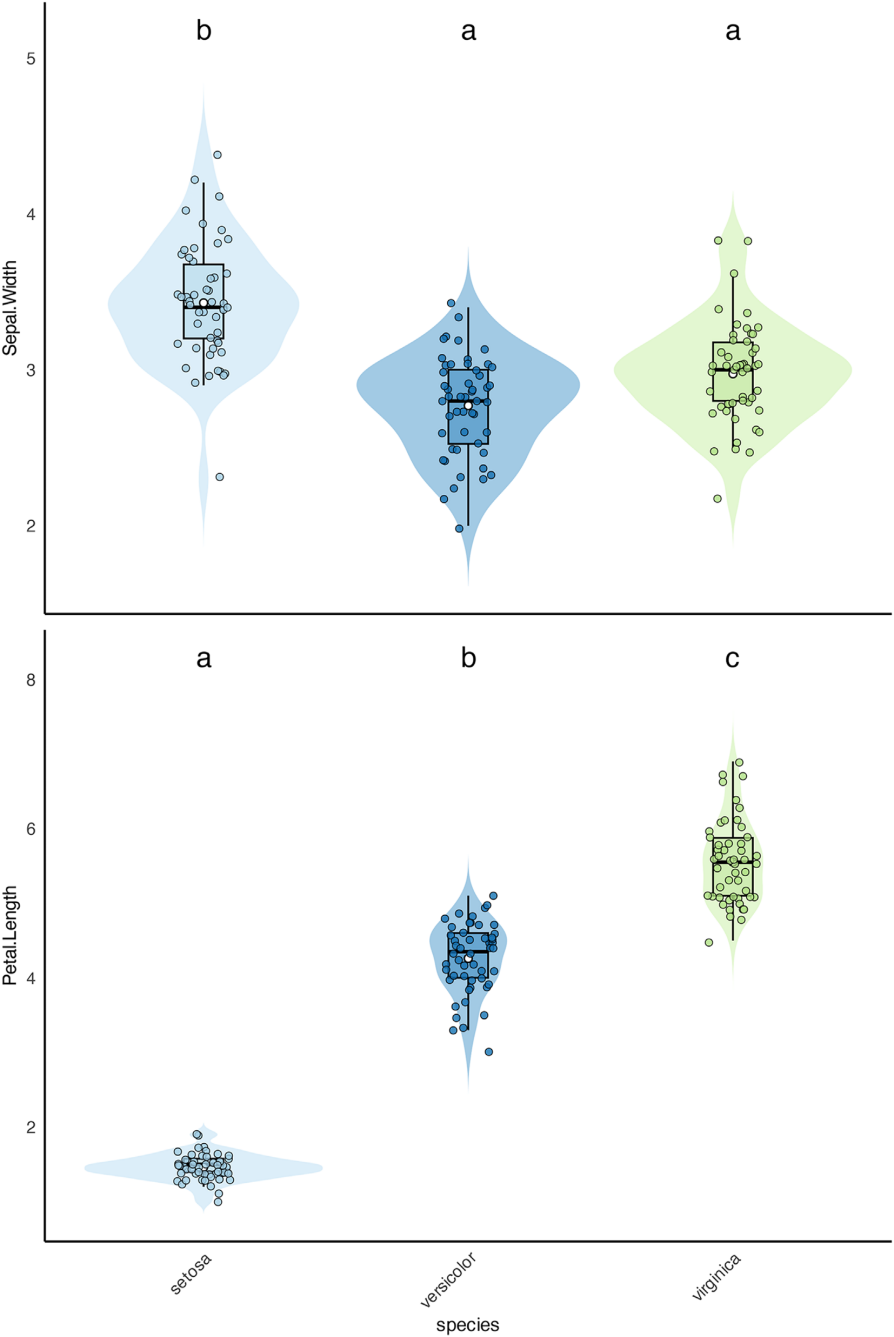
Univariate visualizations using the R “Iris” dataset (no allometric correction required). White dot = mean, horizontal line = median, box = range between 1st and 3rd quantile, top whisker = 1.5 × first 1st, bottom whisker = 1.5 × 3rd quantile (each of the four data quantiles contains 25% of the observations). Violin outlines depict kernel density estimates of trait distributions for each species. Statistical differences are indicated by different letters (post hoc pairwise comparisons). Top depicts the sepal width (ANOVA, *F* = 49.16, Degrees of Freedom between species = 2, Degrees of Freedom within species = 147, *p*‐value = 4.49 × 10^−17^). Bottom depicts petal length (Kruskal–Wallis, Chi squared = 130.41, *p*‐value = 4.80 × 10^−29^).

Univariate analysis identified significant differences among species for several variables across the three datasets. From a total of 31 variables, 14 met the assumptions of normality (Shapiro–Wilk test) and homogeneity of variances (Bartlett's test) and were analyzed using ANOVA. Among these, 13 variables showed significant differences (*p* < 0.05). Post hoc Tukey's Honestly Significant Difference (HSD) tests revealed differences among species. The results, including *F*‐statistics, degrees of freedom, and *p*‐values, were summarized in summary_anova.csv. Corresponding violin and boxplots annotated with Tukey's HSD groupings were generated for visualization (e.g., Figure 4).

For the remaining 17 variables, which did not meet ANOVA assumptions, nonparametric Kruskal–Wallis tests were conducted. Significant differences were identified for 16 variables (*p* < 0.05). Pairwise comparisons were performed using Dunn's test with Bonferroni corrections, revealing significant groupings among species. The results, including Kruskal *p*‐values, Chi‐squared statistics, and significant pairwise comparisons, were saved in summary_kruskalwallis.csv. Annotated violin and boxplots were generated to visualize these differences (e.g., Figure 4).

## 4 Discussion

*Orangutan* provides a comprehensive, user‐friendly framework for conducting phenotypic analyses in a comparative framework, integrating multiple statistical approaches into a single pipeline. By automating data cleaning, outlier identification and removal, allometric correction, univariate and multivariate analyses, and generation of publication‐quality visualizations, *Orangutan* significantly reduces the effort and potential for error commonly encountered in species description workflows (Chan and Grismer 2021).

Our application of *Orangutan* to empirical datasets demonstrates its utility in revealing both clear and subtle phenotypic patterns. The summary statistics function generated a detailed overview of trait variation across species (Table 1), facilitating initial assessments of central tendency and dispersion. In particular, the identification of nonoverlapping diagnostic traits streamlines the process of pinpointing characters that may serve as reliable species identifiers, addressing a critical need in taxonomic practice (Balakrishnan 2005; Cadena and Zapata 2021). These findings underscore the value of automated range‐based comparisons for expediting the discovery of diagnoses, which are often laborious to detect through visual inspection.

Optional allometric correction effectively removed size‐related variation from mensural traits, enabling a focus on shape differences. Subsequent multivariate analyses—PCA and DAPC—revealed coherent species clusters in reduced‐dimensional space (Figure 3), with polygons facilitating the visual delineation of groups of interest (e.g., *A. allisoni* vs. *A. carolinensis* ). The DAPC cross‐classification table (Table 2) demonstrated high assignment accuracy, indicating that phenotypic data alone can robustly predict species identity when proper size correction and discriminant modeling are applied. These results support the combined use of allometric adjustment and classification‐based multivariate methods for species diagnoses.

The univariate hypothesis‐testing framework in *Orangutan* dynamically selects parametric or nonparametric tests based on assumption checks, ensuring appropriate statistical inference for each trait. Boxplots annotated with post hoc group letters provide clear, publication‐ready visual summaries of significant differences. This flexibility enhances reproducibility by standardizing decision criteria for test selection and presentation, alleviating the burden of manual assumption testing and result formatting.

Given the growing interest in integrated software pipelines for phenotypic species diagnosis, it is useful to situate *Orangutan* within the context of existing tools developed for similar purposes. At present, *Orangutan* and GroupStruct2 (Chan and Grismer 2025) represent the only comprehensive pipelines explicitly designed for species diagnosis based on phenotypic data. While both integrate multiple analytical components within a unified framework, they differ fundamentally in analytical philosophy and inferential transparency. *Orangutan* is designed as a reproducible, hypothesis‐driven diagnostic system in which all analytical steps are implemented in a fully scripted, deterministic environment, enabling exact replication, sensitivity analyses, and formal reassessment of analytical decisions. In contrast, GroupStruct2 emphasizes GUI‐mediated, interactive workflows, in which decisions regarding outlier handling, variable inclusion, and group definitions are guided by user input and conducted within an exploratory session‐based framework. Correspondingly, *Orangutan* conditions inference on explicit evaluation of statistical assumptions—such as normality, homoscedasticity, and multivariate dispersion—thereby documenting how these assumptions influence analytical outcomes, whereas GroupStruct2 adopts a more flexible, user‐directed approach consistent with exploratory data analysis.

These philosophical differences are reflected in the analytical emphasis of each framework. *Orangutan* prioritizes hypothesis testing and phenotypic diagnosability of predefined groups, integrating assumption‐aware univariate and multivariate tests with explicit evaluation of effect sizes, nonoverlapping trait ranges, and classification performance. Outlier detection and allometric correction are implemented through rule‐based, reversible procedures with associated diagnostics, allowing users to assess how alternative analytical choices influence variance structure and group separation on a trait‐by‐trait basis. By contrast, GroupStruct2 places greater emphasis on model‐based and ordination‐based approaches, including Bayesian Gaussian mixture models, to explore alternative grouping hypotheses and patterns of morphological structure.

Collectively, these analytical features position *Orangutan* as a stand‐alone framework particularly suited to studies in which reproducibility, explicit assumption testing, and diagnostic robustness are central to species or population comparisons with phenotypic data. By automating key analytical steps and producing high‐quality visual outputs, *Orangutan* enhances the democratization, reproducibility, and efficiency of taxonomic research, ultimately contributing to more accessible and robust species diagnoses and descriptions.

While rooted in Systematics, *Orangutan* could be used to address specific needs of ecologists and evolutionary biologists interpreting complex numeric phenotypic data across varying scales. At the population level, the package's ability to standardize mensural traits and visualize phenotypic overlap offers a robust pipeline for detecting phenotypic plasticity and local adaptation (Schmid and Guillaume 2017). This could allow researchers to distinguish environmentally induced variation from fixed evolutionary differences and quantify morphological divergence between populations facing distinct environmental pressures. Expanding to the community level, the rigorous separation of size from shape through allometric correction enables the precise quantification of functional traits—such as changes in limb proportions across habitat gradients or trophic adaptations in competitive landscapes (Voje et al. 2014). Furthermore, the integrated multivariate workflows could allow researchers to map morphospace occupancy, facilitating quantitative tests of ecological character displacement (Pfennig and Pfennig 2009), niche partitioning (Westeen et al. 2023), and convergent evolution (Speed and Arbuckle 2017). Thus, the package serves as a critical bridge between raw measurements and high‐level ecological inference, ensuring that comparative studies are grounded in reproducible, quantitative evidence.

## Author Contributions

**Javier Torres:** conceptualization (equal), data curation (equal), formal analysis (equal), investigation (equal), methodology (equal), project administration (equal), resources (equal), software (equal), supervision (equal), validation (equal), visualization (equal), writing – original draft (equal), writing – review and editing (equal).

## Conflicts of Interest

The author declares no conflicts of interest.

## Data Availability

The data to reproduce this work and software are freely and publicly available at https://github.com/metalofis/Orangutan‐R, https://cran.r‐project.org/web/packages/Orangutan/index.html and https://zenodo.org/records/18488056.
